# Optimization of Mo/Cr bilayer back contacts for thin-film solar cells

**DOI:** 10.3762/bjnano.9.252

**Published:** 2018-10-18

**Authors:** Nima Khoshsirat, Fawad Ali, Vincent Tiing Tiong, Mojtaba Amjadipour, Hongxia Wang, Mahnaz Shafiei, Nunzio Motta

**Affiliations:** 1 School of Chemistry, Physics, Mechanical Engineering, Queensland University of Technology (QUT), Brisbane, Australia; 2 Swinburne University of Technology, Melbourne, Australia

**Keywords:** back contact, bilayer, chromium, DC sputtering, molybdenum, optical reflectance

## Abstract

Molybdenum (Mo) is the most commonly used material as back contact in thin-film solar cells. Adhesion of Mo film to soda–lime glass (SLG) substrate is crucial to the performance of solar cells. In this study, an optimized bilayer structure made of a thin layer of Mo on an ultra-thin chromium (Cr) adhesion layer is used as the back contact for a copper zinc tin sulfide (CZTS) thin-film solar cell on a SLG substrate. DC magnetron sputtering is used for deposition of Mo and Cr films. The conductivity of Mo/Cr bilayer films, their microstructure and surface morphology are studied at different deposition powers and working pressures. Good adhesion to the SLG substrate has been achieved by means of an ultra-thin Cr layer under the Mo layer. By optimizing the deposition conditions we achieved low surface roughness, high optical reflectance and low sheet resistivity while we could decrease the back contact thickness to 600 nm. That is two thirds to half of the thickness that is currently being used for bilayer and single layer back contact for thin-film solar cells. We demonstrate the excellent properties of Mo/Cr bilayer as back contact of a CZTS solar cell.

## Introduction

Molybdenum (Mo) thin films are widely used as a back contact for photovoltaic devices such as Cu(In_1_*_−x_*Ga*_x_*)S_2_ (CIGS) and Cu_2_ZnSnS_4_ (CZTS) thin-film solar cells. The back contact is the first layer to be deposited and its properties have significant effects on the solar cell performance. This layer acts as an optical reflector to the photons that are not absorbed in the active medium, and as a metallic contact layer to transport drive out the photo-generated carriers [1–2]. In addition to these electro-optical properties, the back contact material should have a higher work function than the neighboring light-absorbing semiconductor layer [3]. Accordingly, different metal contacts (such as Al, Au, Cr, Mn, Mo, Pt, Ti, V and W) have been used as back contact layer in thin-film solar cells [4–7]. Among these elements, Mo is reported to have a relatively better stability at the elevated temperatures required for the fabrication of CIGS and CZTS, better ohmic contact behavior, lower resistivity and a higher work function than the CIGS and CZTS light-absorbing semiconductor layers. However, the most challenging part in the deposition of the Mo thin film using a DC sputter system is to find the right compromise between the adhesion of the film to the substrate (mostly soda–lime glass (SLG)) and its electro-optical properties. It is well known that the film adhesion and its conductivity show opposite trends as a functions of the sputtering power and pressure [8–9]. The most successful approach suggested so far to improve the adhesion of Mo back contact layer to the glass substrate (while retaining its conductivity and optical reflectance) is through the deposition of a Mo bilayer rather than a single layer [10–13]. This involves the deposition of a thick Mo layer with low resistivity at low pressure and high sputtering power on a thin Mo layer deposited at high pressure and low power providing the required adhesion to the substrate. However, this method still does not guarantee the adhesion of the film to the substrate and the Mo back contact layer could peel off from the substrate during the absorber layer growth at high temperatures or during the deposition of the buffer layer, which most commonly involves a wet chemical process. Moreover, the thickness of the bilayer Mo film needs to be in the range of 900 nm to 1.2 μm to achieve a good conductivity. One of our goals in this work was to reduce the back contact thickness, which would lead to cost reduction [14–16].

Cr is a well-known adhesion layer, traditionally used to increase the adhesion of metallic films to substrates such as glass or stainless steel [17–21]. Cr forms an oxide interface layer by scavenging the oxygen available on the glass surface during the sputtering process. This creates nucleation centers and promotes nucleation of the next deposited material (in this case Mo), leading to a strong adhesion of Mo to the substrates [22–24]. The Mo/Cr back contact has been already proposed in flexible thin-film solar cells on metallic foils and stainless steel [25–28]. However, in these reports Cr was used as a barrier layer to reduce/prevent impurity out-diffusion from the metallic substrate to the absorber layer and its effect on the adhesion of the back contact was not studied. In spite of the importance of this topic for the development of thin-film solar cells, there are very few reports regarding the application of Cr as an adhesion layer in back contacts [29–30]. Notably, the influence of Cr on the properties of the back contact and on the total performance of the solar cell is still a subject of debate and some studies negatively correlates Cr with a low performance of the cell due to its diffusion in the absorber layer [31]. In this work we exploit the adhesive properties of Cr to develop a new protocol that improves the adhesion of the Mo back contact layer to the substrate and demonstrate the excellent electro-optical properties of the Mo/Cr film for solar cells applications. The morphological and optical properties of the deposited films have been investigated using scanning electron microscopy (SEM), atomic force microscopy (AFM), UV–vis–NIR spectroscopy and X-ray photoelectron spectroscopy. A careful analysis of the resulting Mo/Cr thin film across all the sputtering parameters led us to the best combination, optimizing both the electro-optical response of the Mo/Cr bilayer and the adhesion of the film to the substrate while we could reduce the required thickness to 600 nm. That is at most two thirds of reported thickness for Mo back contacts for thin-film solar cells.

## Experimental

### Film deposition

A 10–15 nm thick layer of Cr was deposited on a 2.5 × 2.5 cm SLG substrate using a Kurt J. Lesker PVD75 DC magnetron sputtering system operated at 40 W and 10 mTorr for 10 min, from a 2 inch Cr target (Kurt J. Lesker, 99.95% purity). The Mo layer was deposited on uncoated and Cr-coated substrates by using a 2 inch Mo target (Kurt J. Lesker, 99.95% purity) at different working pressures (3, 5 and 10 mTorr) and different sputtering powers of (100, 150 and 200 W). In order to start under the same conditions for all depositions, the vacuum chamber was evacuated before each deposition round to the base pressure of 1 × 10^−7^ Torr, and the targets were pre-sputtered for 10 min with Argon in order to remove any oxide layer or contamination. During the deposition, the substrates were continuously rotating with a speed of 20 rpm to ensure a homogeneous coverage all over the substrate surface. The sputtering time was determined from the deposition rate under different working conditions and was set to obtain Mo layers with 600 nm thickness.

### Film characterization

The film thickness measurements were conducted using a Bruker Dektak stylus profiler. Morphology studies and surface roughness measurements were carried out using a JEOL 7001F scanning electron microscope (SEM) and a tapping mode NT-MDT Solver-Pro atomic force microscope (AFM). A KeithLink four-point probe system was used to measure the sheet resistivity of the films. A Cary 5000 UV–vis–NIR spectrophotometer was also used for the optical properties measurements. The adhesion of the Mo layer was tested through ultra-sonication of the samples at 50 °C in the presence of sodium hydroxide solution (2 wt % in deionized water) for 10 min. The crystal structure of Mo films has been analyzed using a Rigaku SmartLab X-ray diffraction (XRD) spectrometer with monochromatic Cu Kα (40 kV, 40 mA, λ = 0.154 nm) as an excitation source operating in parallel beam mode with a Hypix 3000 detector (0D mode). The incidence angle (α) was fixed to 1° during data collection. The incident optics were a 5° Soller slit, 10 mm incident slit and a 0.137 mm divergence slit. Receiving optics were a 0.114° collimator and 20 mm receiving slits. Patterns were collected for 1 h at a step size of 0.01° from 10 to 85° 2θ at 1.3° per minute over the 2θ axis.

X-ray photoelectron spectroscopy (XPS) was performed using a Kratos Axis Supra with Al Kα X-ray radiation (*h*ν 1486.7 eV). High-resolution scans of the O 1s, C 1s, Na 1s, Cr 2p, Na 1s and Mo 3d regions were acquired with 10 eV pass energy and about 0.4 eV spectral resolution to discriminate the substructure of the spectral lines. Ar^+^ ion cluster etching was employed for XPS depth profiling and the calibration was carried out using a typical Mo on glass sample with sputtering rate of 15.5 nm/s. Depth profiling was conducted on a 1 × 1 mm^2^ area with a Ar^+^ ion beam energy of 10 keV and large cluster size (*n* = 2000).

## Results and Discussion

### Adhesion

The first characterization step was the adhesion test performed on Mo layers after deposition on uncoated and Cr-coated glass substrates. All samples deposited on uncoated glass showed poor adhesion to the substrate, while a thin layer of Cr significantly improved the adhesion of Mo layer to the substrate (see Supporting Information File 1).

**Figure 1:**
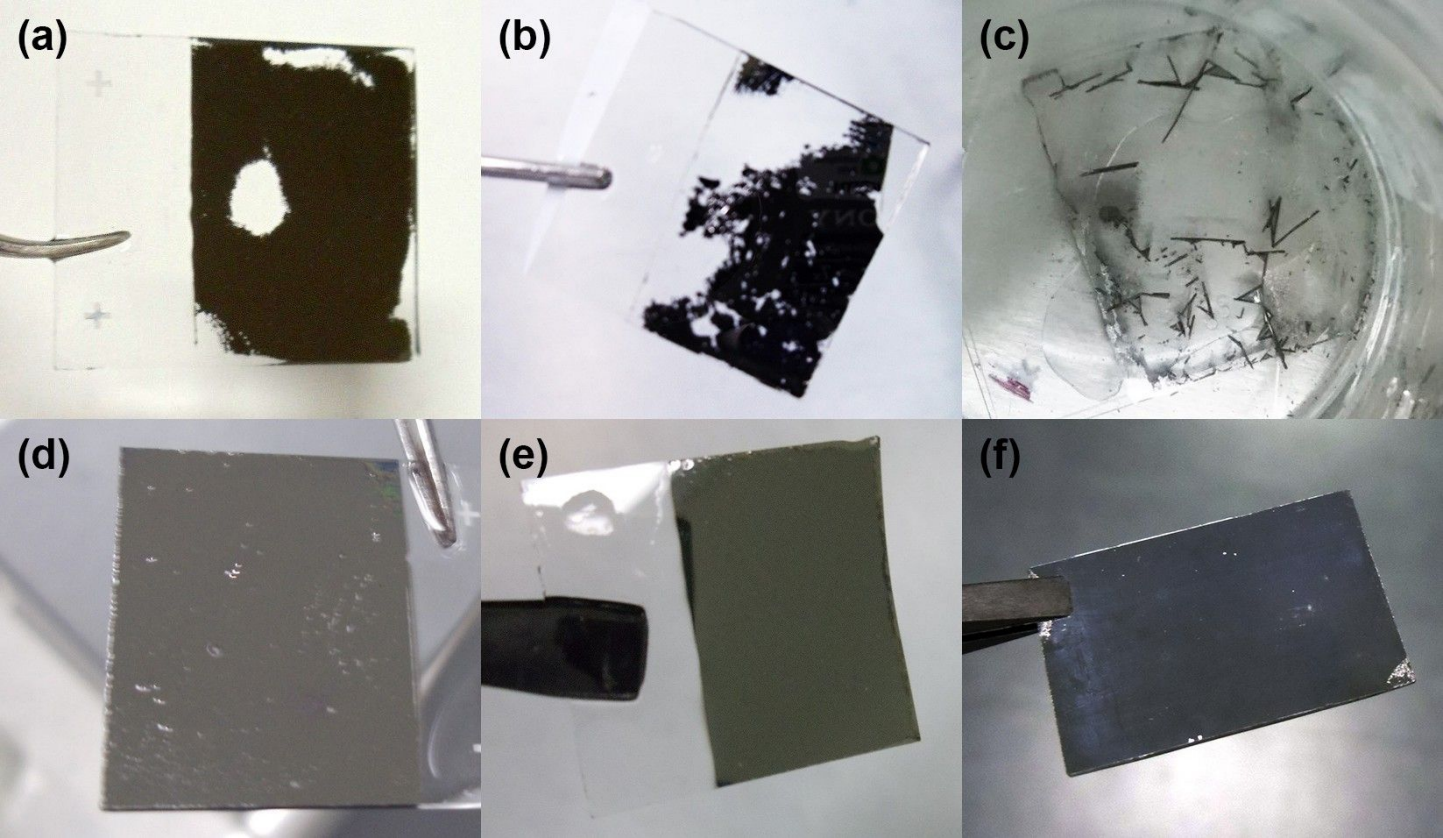
Samples after adhesion test. (a–c) Mo/SLG; (d–f) Mo/Cr/SLG. (a, d) 100 W, 10 mTorr, (b, e) 150 W, 5 mTorr, (c, f) 200 W, 3 mTorr.

Figure 1 shows pictures of some samples after the adhesion test. It is evident how the Mo layer is peeling off from the uncoated glass substrate (Figure 1a–c) failing the adhesion test, while the same layer deposited on Cr-coated glass substrates does not peel off (Figure 1d–f).

### Resistivity

**Figure 2:**
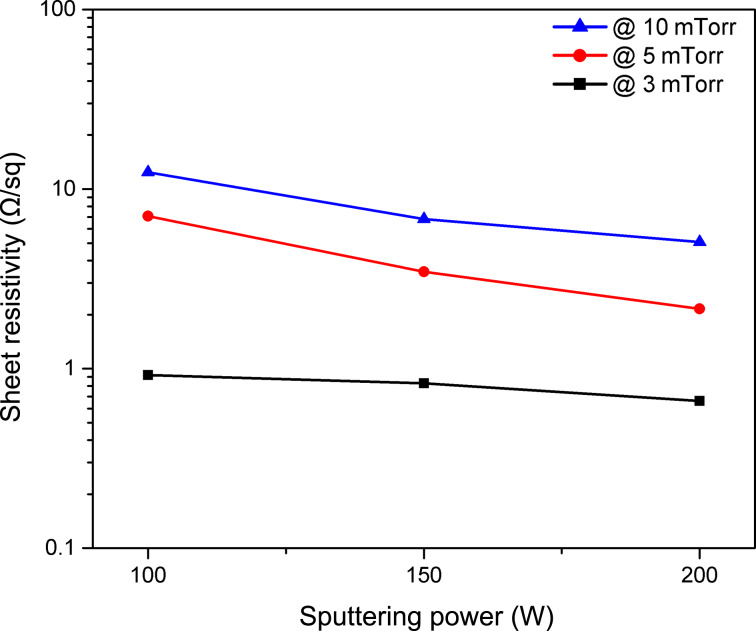
Sheet resistivity of Mo/Cr films prepared at different values of sputtering power and pressure.

Besides the good adhesion to the substrate, the metallic back contact layer in thin-film solar cells should have low resistivity to be able to drive out the photo-generated carriers to the external load. Sheet resistivity for a typical back contact layer should not be larger than 1 Ω/sq. Measurements using a four-point probe were performed only on samples that passed the adhesion test. Figure 2 shows the variation of Mo/Cr bilayer electrical resistivity at different deposition pressures with respect to the sputtering power. As can be seen, the resistivity of Mo/Cr bilayer is directly proportional to the working pressure, while it shows an inverse relation to the sputtering power. Accordingly, the resistivity of the layers reduces by increasing the sputtering power. The decrease in resistivity due to the increase of sputtering power is more drastic at 10 and 5 mTorr compared to the samples prepared at 3 mTorr. Samples deposited at 10 mTorr show high sheet resistivity, even at high sputtering power (5.07 Ω/sq = 304.2 μΩ·cm at 200 W), unsuitable for back contacts of thin-film solar cells. The lowest resistivity is shown by films prepared at 3 mTorr and 200 W (0.66 Ω/sq = 39.6 μΩ·cm), falling in the required range for back contacts. This range of conductivity has been reported for bilayer Mo films with thickness values in the range of 0.9 to 1.2 μm [14–16], which is 1.5-times the value of our samples.

**Figure 3:**
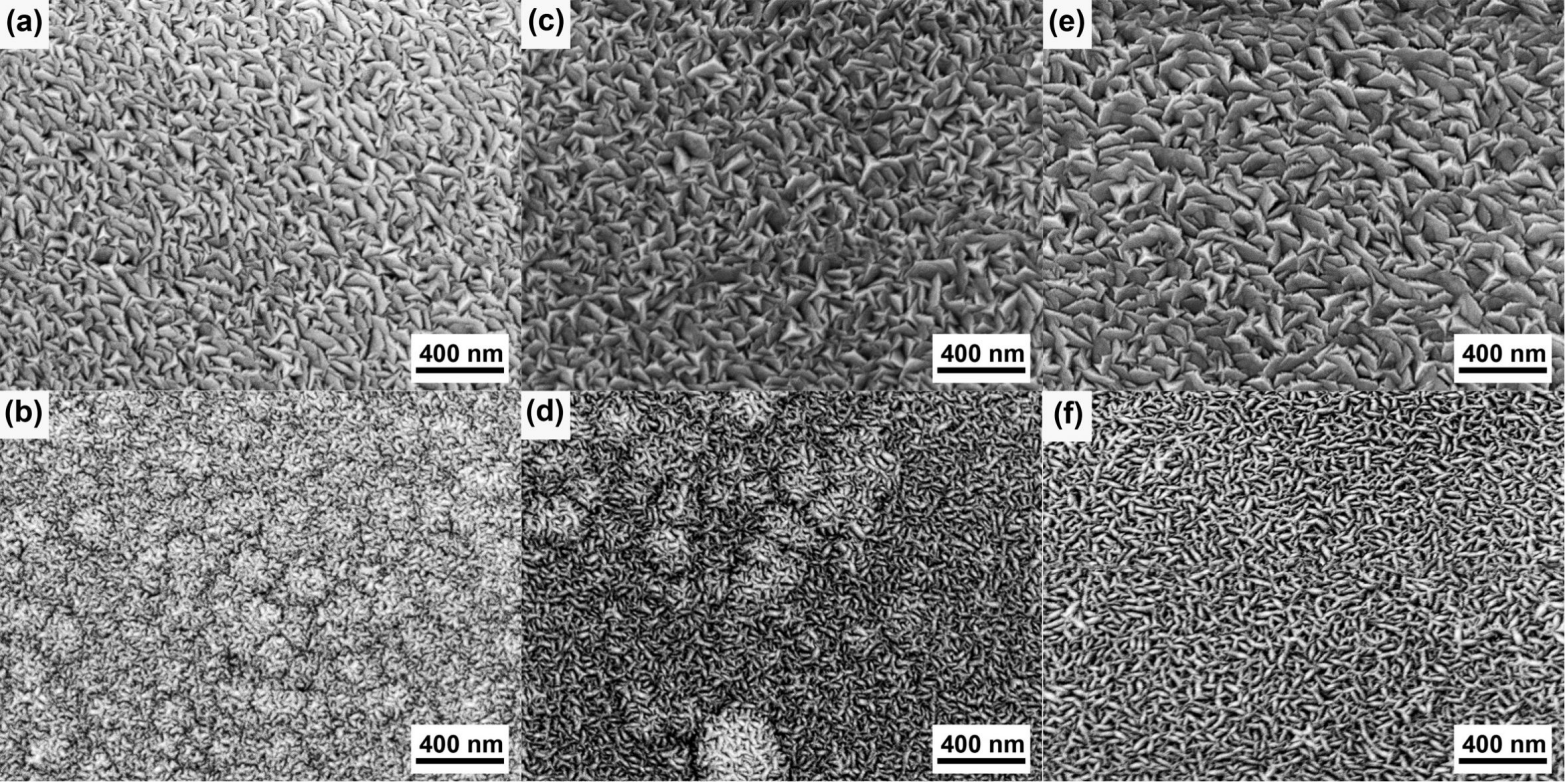
Surface SEM images of Mo/Cr films prepared at sputtering power and pressure values of (a) 100 W, 3 mTorr, (b) 100 W, 5 mTorr, (c) 150 W, 3 mTorr, (d) 150 W, 5 mTorr, (e) 200 W, 3 mTorr and (f) 200 W, 5 mTorr.

The decreasing of the film resistivity with increasing sputtering power could be attributed to the effect of power on the microstructure of the film. Higher power leads to bigger grain sizes, as higher kinetic energies of the atoms favor the coalescence of grains. Similarly, low pressure leads to higher grain sizes due to the lower number of collisions and the higher energy of atoms landing on the substrate [9,32–33]. As shown in Figure 3, the films deposited at lower pressure and higher power have larger and more dense grains as compared with the films deposited at higher pressure and lower power. This results in less grain boundaries and consequently higher carrier mobility and conductivity.

**Figure 4:**
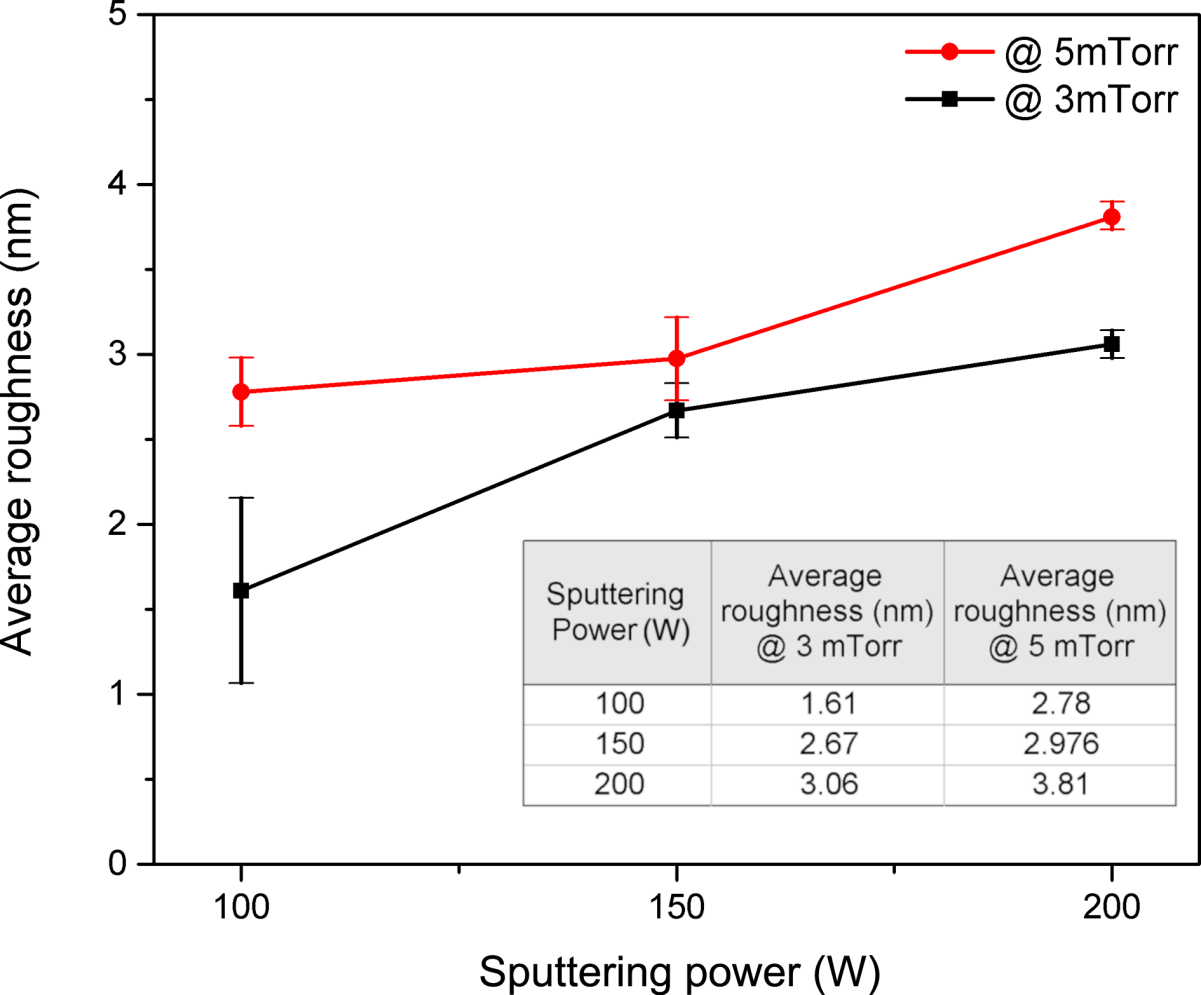
Average surface roughness of Mo/Cr films prepared at different sputtering powers and pressures.

The SEM images also show more uniform surfaces over the film area for the samples deposited at 3 mTorr compared with those films deposited at 5 mTorr. Blister-shaped features appeared on the samples deposited at 5 mTorr possibly due to micro-bubbles of Ar gas trapped in the Mo layer [34]. The appearance of blisters on the sample surface decreases when the sputtering power is increased. The differences in surface morphology are also confirmed by AFM images (see Supporting Information File 1). AFM roughness analysis (Figure 4) reveals an increase of the surface roughness with the sputtering power. Surface roughness is also directly proportional to the working pressure. The average roughness increases from 1.61 to 3.06 nm in samples deposited at 3 mTorr and from 2.78 to 3.81 nm in samples deposited at 5 mTorr, when the power is raised from 100 to 200 W, confirming the grain size increase visible in the SEM results (Figure 3), as higher power leads to denser and bigger grains.

### X-ray diffraction (XRD) pattern and crystallinity

**Figure 5:**
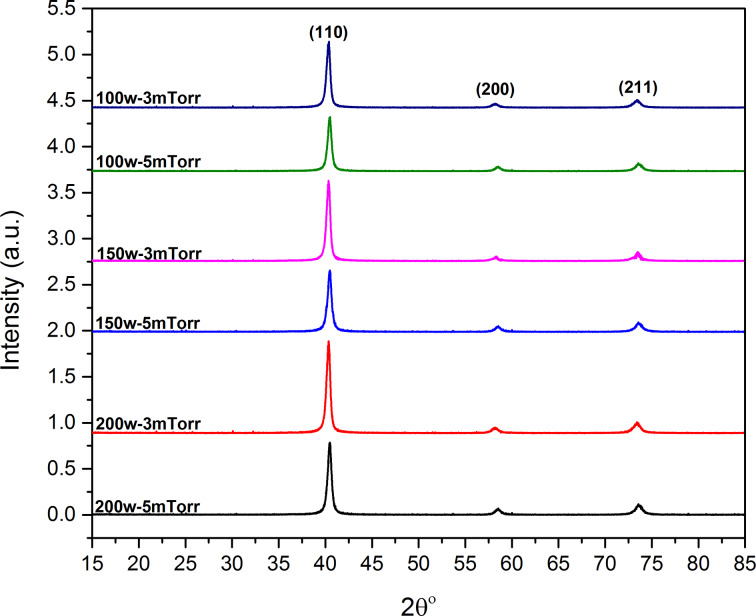
X-ray diffraction peaks of Mo/Cr films prepared using different values of sputtering power and pressure.

The XRD spectra of Mo films grown on Cr-coated glass using different values of sputtering power and pressure are shown in Figure 5. All samples exhibit three main peaks that match with the (110), (200) and (211) plane orientations of the standard difractogram ICDD-96-901-1606 for Mo. A comparison of XRD peak intensities shows a higher peak intensity for samples deposited at 3 mTorr. This is another evidence for increased crystallinity at lower deposition pressure. A similar trend can also be seen in Figure 5 when the sputtering power increases from 100 to 200 W.

XRD patterns of all samples exhibit a dominant (110) orientation. Previous studies revealed that the (110) Mo crystal orientation can enhance the (220) and (204) CIGS and (112) CZTS orientations, which leads to a decrease of grain boundary recombination losses and series resistance in CIGS and CZTS thin-film solar cells [35–36]. Table 1 shows the summary of the analysis of the (110) peaks. The FWHM values of the samples prepared at lower pressure are lower, which also confirms the presence of larger crystalline grains at low deposition pressures.

**Table 1:** X-ray diffraction analysis of peak along the (110) plane.

sample	(110) peak FWHM	(110) peak position	110/(all peaks)
	±0.001	±0.01	

100W-5mTorr	0.450	40.454°	0.75
150W-5mTorr	0.478	40.452°	0.74
200W-5mTorr	0.451	40.453°	0.74
100W-3mTorr	0.430	40.326°	0.77
150W-3mTorr	0.431	40.325°	0.81
200W-3mTorr	0.430	40.325°	0.77

### Optical properties

**Figure 6:**
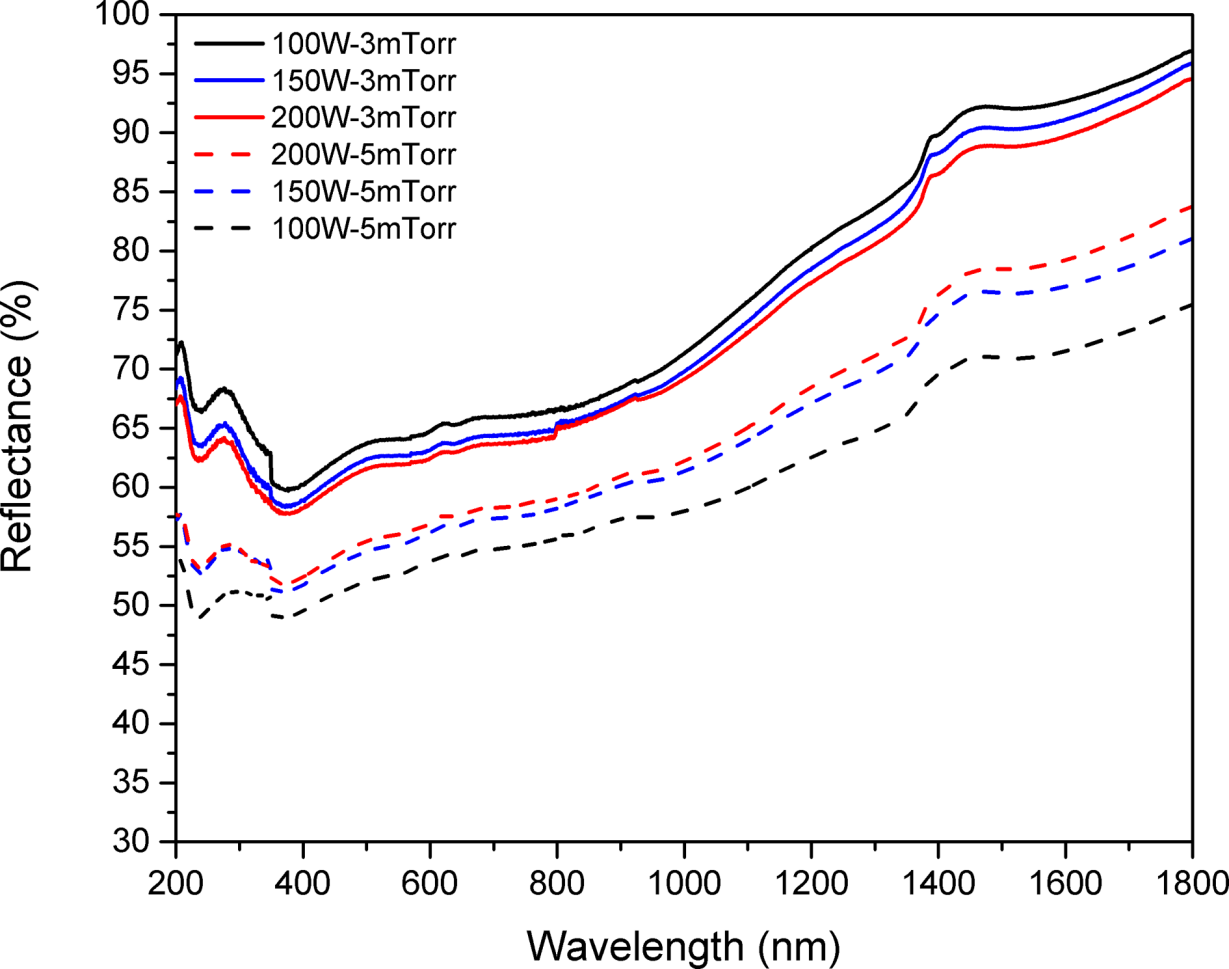
Optical reflectance of Mo/Cr films prepared using different values of sputtering power and pressure.

Another important characteristic of a good back contact layer is its optical reflectance. A back contact layer with high optical reflectance can contribute effectively to the light absorption by reflecting the non-absorbed photons back to the absorber layer. The optical reflectance of the the Mo/Cr bilayer was measured in the wavelength range of 200–1800 nm. As shown in Figure 6, films deposited at 3 mTorr have higher reflectance than those deposited at 5 mTorr. UV–visible spectroscopy results also revealed that the optical reflectance of the films slightly decreases after increasing the sputtering power, due to the roughness increase. In order to quantify the reflectance variation due to changes in deposition power and pressure, the area under each reflectance curve was calculated by numerical integration and called integral reflectance. Integral reflectance shows a 1.38% increase when the sputtering power decreases from 200 to 150 W and a 2.21% further increase when the power decreases to 100 W, for samples deposited at 3 mTorr, in general agreement with reports on single-layer or bilayer Mo films [4,37–39]. It should be noted however, that our DC-sputtered Mo/Cr bilayer shows an overall optical reflectance of 60–65% in the visible range, which is much higher than previously reported sputtered Mo films (35–60%) [8–10,37–40]. This is mostly due to lower surface roughness of our samples.

### Diffusion of chromium and sodium

**Figure 7:**
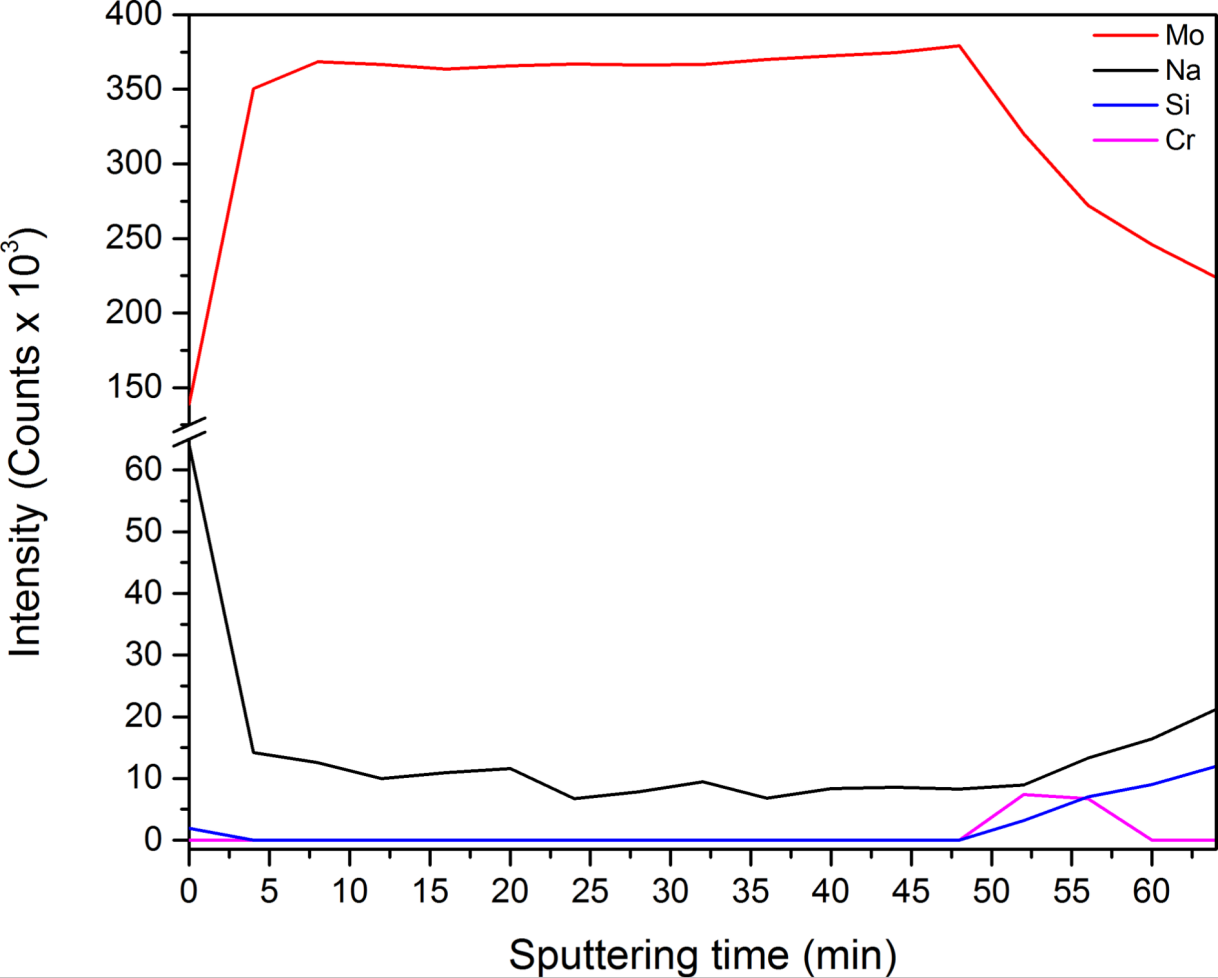
XPS depth profile of the Mo/Cr films deposited at 200 W and 3 mTorr.

It is known that in CIGS and CZTS thin-film solar cells diffusion of sodium from the SLG substrate to the absorber layer is beneficial and can enhance the electro-optical properties of the absorber layer [41–45]. On the other side, Cr incorporation in CIGS absorber layer reduces the cell performance due to creation of deep defect levels in the CIGS layer [46]. In order to investigate the diffusion mechanism of Cr and Na to the top layer, we first heated the Mo/Cr films on SLG to 550 °C for 30 min in argon atmosphere. This is the temperature that is normally used for sulfurization and selenization of CIGS and CZTS layers. Then XPS depth profiling was performed on the annealed Mo/Cr films to search for any Cr or Na signal in the Mo layer, by etching the film via argon gas cluster ions for 4 min before each XPS acquisition until the signal from the substrate was visible. The result is shown in Figure 7 for a Mo/Cr film deposited at 200 W and 3 mTorr (high-resolution spectra of Mo 3d and Cr 2p are provided in Supporting Information File 1).

The profile clearly shows the presence of sodium throughout Mo layer and even on the surface which means that the thin Cr adhesion layer is not stopping Na diffusion from SLG to the top layers. No Cr signal has been detected in the Mo layer. Cr signal starts to increase at 50 min sputtering, exactly when the Mo signal starts to drop, and then drops to zero at around 60 min, confirming that Cr does not diffuse in the Mo layer and is just acting as an adhesion layer.

### Applying the developed Mo/Cr bilayer in a CZTS thin-film solar cell

**Figure 8:**
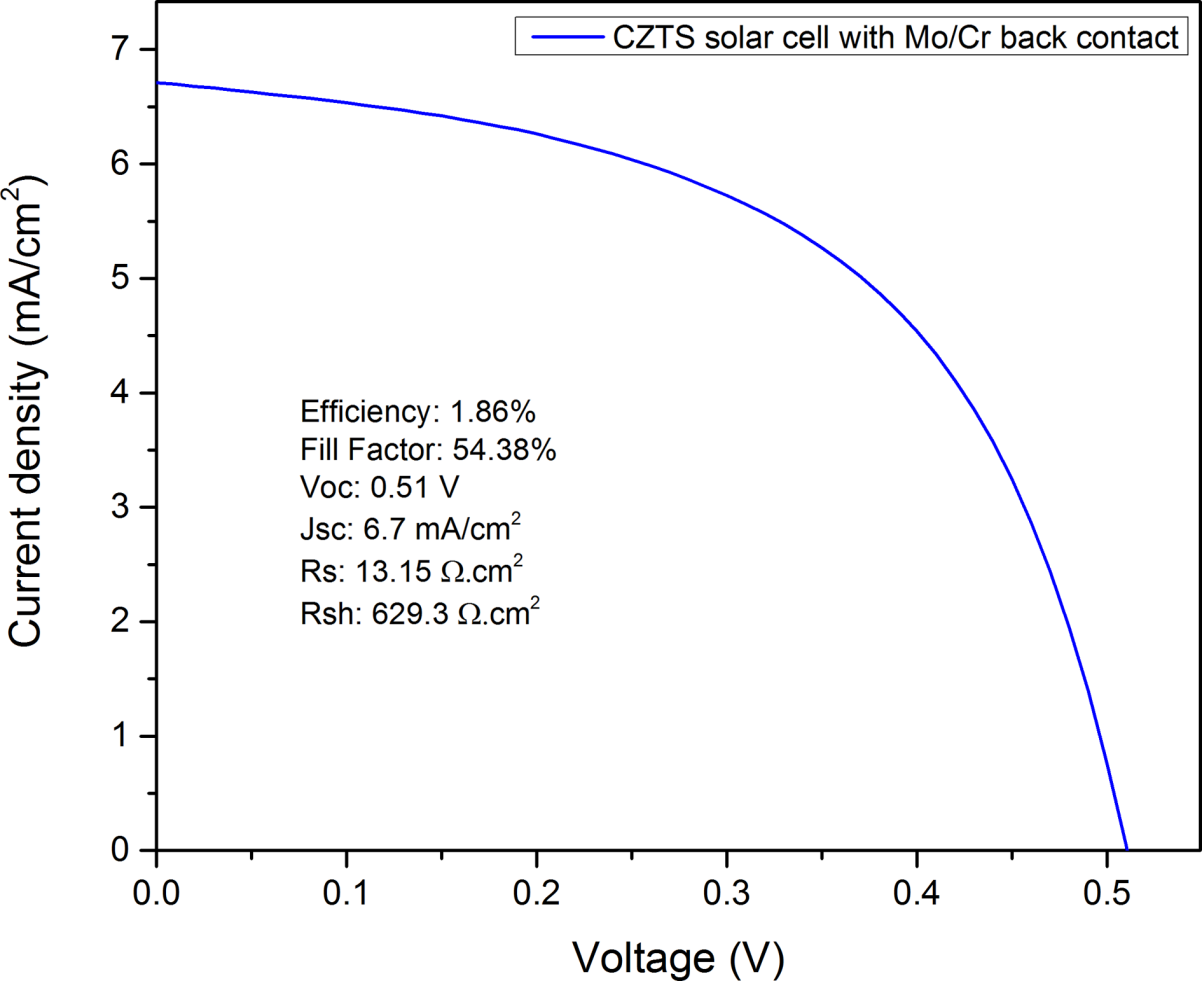
*J*–*V* characteristics of the CZTS cell with Mo/Cr bilayer back contact.

The bilayer Mo/Cr stacks developed in this work seems to be a good option to be used as a back contact in thin-film solar cells. In order to prove this a CZTS thin-film solar cell has been made by stacking the ITO/ZnO/CdS/Cu_2_ZnSnS_4_ on top of Mo/Cr bilayer back contact on the SLG substrate. A 1.5 μm thick CZTS was deposited through a two-step process of sulfurization of stacked metallic layers of Cu/Sn/Zn. Then a 60 nm CdS buffer layer was deposited using chemical bath deposition (CBD). This was followed by sputtering of a 30 nm ZnO layer and a 350 nm ITO layer as transparent conductive oxide (TCO) layers. As the last step, a silver collection grid was deposited on top using electron-beam evaporation. It is worth mentioning that the fabrication of a cell on a single-layer Mo-coated glass also has been tried. However, the Mo films were peeled-off from the substrate during CdS CBD. Thus, herein only the performance of a cell with Mo/Cr bilayer back contact is reported. Figure 8 shows the *J*–*V* characteristics of the CZTS cell with Mo/Cr bilayer back contact. In this first attempt it is clear that the device is working with a reasonable fill factor (54.38%). The efficiency of the cell was 1.86%, calculated over an area of 0.173 cm^2^. This measurement confirms the functionality of the Mo/Cr bilayer back contact for CZTS thin-film solar cells. A substantial improvement of the performance is expected to come through an optimization of the electro-optical properties of the layers, which is the subject of further studies.

## Conclusion

A bilayer of Mo/Cr thin films with robust adhesion and desired electro-optical properties as a back contact in thin-film solar cells was successfully deposited on SLG substrates using DC sputtering. The bottom 10–15 nm thick Cr layer was used to increase the adhesion of the top Mo layer to the substrate. The experimental results indicate that the Cr layer can significantly improve the adhesion of Mo layer to the glass substrate even for samples that are deposited at high power and low pressure. The lowest electrical resistivity (0.66 Ω/sq ) was observed for a sample with Mo layer deposited at 200 W sputtering power and 3 mTorr working pressure. The optical reflectance in the visible range was found to be over 60% for samples deposited at 3 mTorr. The excellent adhesion of Mo/Cr bilayer to the substrate, its low resistivity and high optical reflectance achieved in this study are matching the requirements for an optimum back contact in CIGS and CZTS thin-film solar cells. The developed Mo/Cr bilayer has been tried in a CZTS thin-film solar cells showing promising values of fill factor and series resistance.

## Supporting Information

File 1 Additional experimental data.
