# Supplementary material for: Optimization of Mo/Cr bilayer back contacts for thin-film solar cells
Source: Beilstein J Nanotechnol. 2018 Oct 18;9:2700–7. doi: 10.3762/bjnano.9.252 (PMC6204819; doi:10.3762/bjnano.9.252)
Supplement: File 1 — Additional experimental data. [file Beilstein_J_Nanotechnol-09-2700-s001.pdf]

Supporting Information

for

Optimization of Mo/Cr bilayer back contacts for thin-film solar cells

Nima Khoshsirat[1], Fawad Ali[1], Vincent Tiing Tiong[1], Mojtaba Amjadipour[1], Hongxia Wang[1], Mahnaz Shafiei[1,2] and Nunzio Motta[*1]

Address: [1]School of Chemistry, Physics, Mechanical Engineering, Queensland University of Technology (QUT), Brisbane, Australia and [2]Swinburne University of Technology, Melbourne, Australia

Email: Nunzio Motta - n.motta@qut.edu.au

[*] Corresponding author

Additional experimental data

Summary of adhesion test results

Table S1: Adhesion test performed on 600 nm thick Mo layers, sputtered at different power and pressure on uncoated and Cr-coated substrates. Cr thickness is 10–15 nm.

Substrate	Power (W)	Pressure (mTorr)	Adhesion Test
Uncoated	100	10	Failed
Uncoated	150	10	Failed
Uncoated	200	10	Failed
Uncoated	100	5	Failed
Uncoated	150	5	Failed
Uncoated	200	5	Failed
Uncoated	100	3	Failed
Uncoated	150	3	Failed
Uncoated	200	3	Failed
Coated	100	10	Pass
Coated	150	10	Pass
Coated	200	10	Pass
Coated	100	5	Pass
Coated	150	5	Pass
Coated	200	5	Pass
Coated	100	3	Pass
Coated	150	3	Pass
Coated	200	3	Pass

Surface AFM images of Mo/Cr films

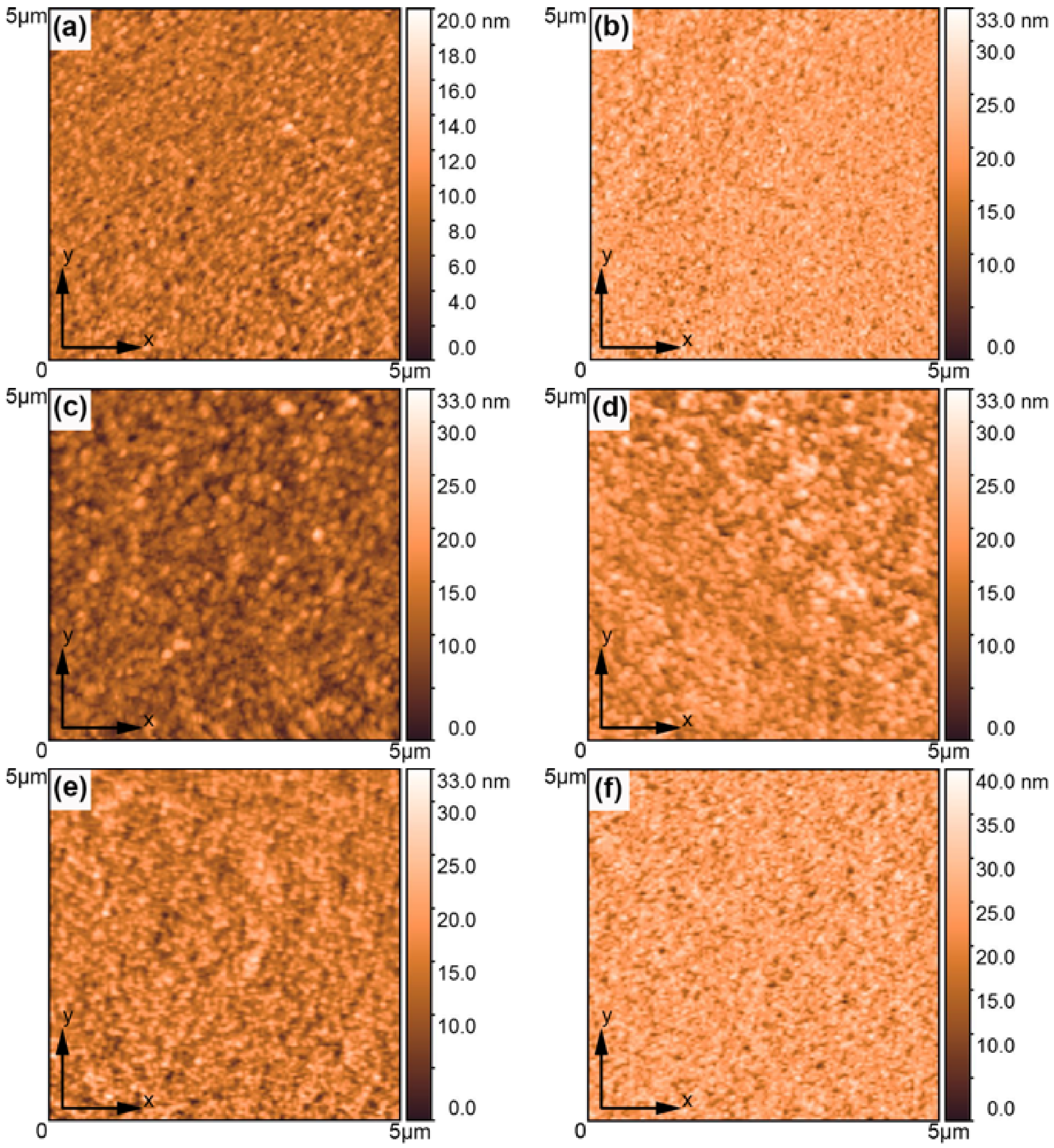

Figure S1: Surface AFM images of Mo/Cr films prepared at sputtering power and pressure of (a) 100 W, 3 mTorr, (b) 100 W, 5 mTorr, (c) 150 W, 3 mTorr, (d) 150 W, 5 mTorr, (e) 200 W, 3 mTorr, (f) 200 W, 5 mTorr.

High-resolution XPS measurements

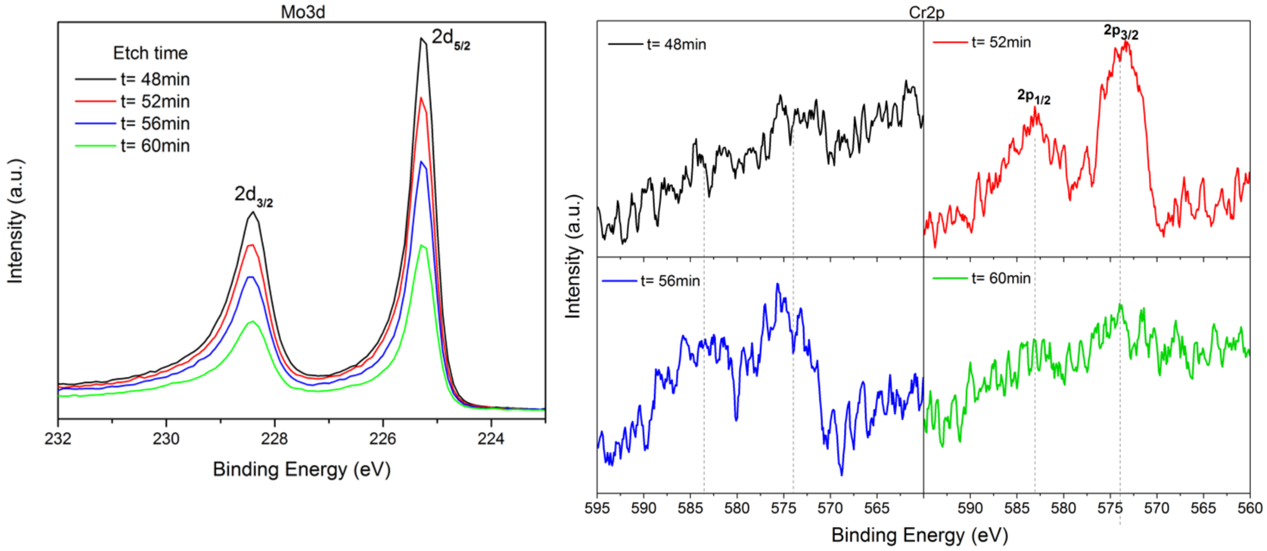

Figure S2: High-resolution XPS scans of Mo 3d and Cr 2p at the Mo/Cr interface.
